# An ARVC-5 *Drosophila* knock-in model reveals new functions of Tmem43 in lipid homeostasis

**DOI:** 10.1242/bio.062326

**Published:** 2026-04-15

**Authors:** Kai Jürgens, Lena Menzel, Nora Klinke, Lisa Schäper, Sandra Ratnavadivel, Stefan Walter, Hendrik Milting, Heiko Meyer, Achim Paululat

**Affiliations:** ^1^ Faculty of Biology and Chemistry, Zoology and Developmental Biology, University of Osnabrück, Barbarastraße 11, 49076 Osnabrück, Germany; ^2^ Erich and Hanna Klessmann Institute, Heart and Diabetes Center NRW, University Hospital of the Ruhr-University Bochum, Georgstraße 11, 32545 Bad Oeynhausen, Germany; ^3^ Center of Cellular Nanoanalytics Osnabrück (CellNanOs), University of Osnabrück, Barbarastraße 11, 49076 Osnabrück, Germany

**Keywords:** *Drosophila*, ARVC-5, ER-mitochondrial contact site, Mitochondria, Cardiomyopathy

## Abstract

Arrhythmogenic right ventricular cardiomyopathy type 5 is caused by the missense mutation S358L in the gene *TMEM43* in humans. To date, the molecular mechanisms underlying the disease remain poorly understood. We established a CRISPR/Cas9 knock-in *Drosophila* model carrying the orthologous *Tmem43^p.S333L^* mutation to investigate these mechanisms *in vivo*. The resulting flies were viable but displayed reduced lifespan, smaller body size, lipid droplet accumulation, and mitochondrial defects. Proteomic and lipidomic profiling revealed a dosage-dependent misregulation of the energy metabolism, concomitant with reduced fatty acid synthesis and ß-oxidation rates, altered peroxisomal pathways, and changes in membrane phospholipid composition. Notably, phosphatidylethanolamine (PE) and phosphatidylinositol (PI) levels were elevated, while triacylglycerols were reduced. Ultrastructural analyses confirmed mitochondrial degradation in the muscle tissue of corresponding mutants. These findings establish Tmem43^p.S333L^ knock-in flies as a robust *in vivo* model of ARVC-5 and support a role for TMEM43 in linking lipid homeostasis to mitochondrial energy metabolism and integrity. Mutation-derived impairments in these processes result in cardiomyopathy.

## Teaser

**Summary:** Tmem43^p.S333L^ mutant flies exhibit shortened lifespan, reduced size, lipid droplet accumulation, mitochondrial defects, and dose-dependent disruption of energy metabolism and lipid homeostasis, linking TMEM43 to cardiomyopathy *in vivo*.

## INTRODUCTION

Arrhythmogenic right ventricular cardiomyopathy (ARVC) is a genetic disorder that commonly involves fibrofatty replacement of cardiomyocytes. Clinically relevant effects include ventricular tachycardia, heart failure, and sudden cardiac death (SCD). The disease can affect both ventricles and is also referred to in the literature as arrhythmogenic cardiomyopathy (ACM). ARVC type 5 (ARVC-5) is a rare subtype associated with the *TMEM43* gene (cytogenetic location 3p25.1). *TMEM43* encodes the protein TMEM43, also called LUMA, which was first identified in a proteome study on nuclear rim proteins (Dreger et al., 2001). Further analyses suggest that TMEM43 localises to the endoplasmic/sarcoplasmic reticulum (ER/SR) and consists of four transmembrane domains (TMDs) and an acidic loop of currently unknown function between TMD 1 and TMD 2, the latter facing the ER lumen (Bengtsson and Otto, 2008). The missense mutation p.S358L, which localises to TMD 3 in TMEM43, causes the clinical diagnosis of ARVC-5 and is fully penetrant in male carriers. Disease manifestations include cardiac arrhythmia, heart failure, and sudden cardiac death. Median life expectancy is 41 years in affected males compared to 71 years in affected females (Merner et al., 2008). To better understand the molecular and physiological causes of TMEM43^p.S358L^ derived ARVC-5, several animal models have been established in recent years. Since TMEM43 is highly conserved across metazoa, we could replicate the clinically relevant missense mutation in the corresponding *Drosophila* gene Tmem43^p.S333L^ (formerly CG8111) and thus take advantage of the experimental possibilities provided by *Drosophila* as a disease model. Transgenic *Drosophila* lines overexpressing the mutated Tmem43 protein exclusively in cardiomyocytes develop arrhythmias and cardiac arrest, thereby mimicking the clinical pathology of patient carriers (Klinke et al., 2022). Interestingly, if the mutated Tmem43 protein is expressed ubiquitously, the induced phenotypes go beyond these cardiac effects. Such animals develop systemic defects, with larvae exhibiting reduced size and weight, concomitant with early adult lethality. Proteome and metabolome analyses indicated that the mutated Tmem43 impairs mitochondrial energy metabolism, among other essential cellular pathways (Klinke et al., 2022). More recent analyses revealed that the Tmem43^p.S333L^ mutation in flies impairs the Tmem43/Porin interaction, which, in turn, destabilises ER/SR-mitochondrial contact sites (ERMCSs). The resulting lack of organelle communication eventually leads to degenerating mitochondria that lose their membrane potential and become non-functional (Jürgens et al., 2025). Similar effects were also observed in human induced pluripotent stem cell (hiPSC) derived cardiomyocytes (Ratnavadivel et al., 2026).

A correlation between TMEM43 and mitochondrial energy metabolism has also been confirmed in other TMEM43 disease models. A transcriptome analysis of cardiac tissue from mice identified a dysregulation of several metabolism-related pathways, and these findings were confirmed in a knock-in mouse model carrying the TMEM43^p.S358L^ mutation. This led to the suggestion that TMEM43 might also play a significant role in fat absorption, metabolism, and storage, particularly under fluctuating environmental conditions such as dietary shortages (Gu et al., 2022). This suggests that the functions of TMEM43 are associated with fundamental cell biological processes, which are presumably conserved in *Drosophila* and vertebrate hearts, such as mitochondrial energy production.

Up to now, the *Drosophila* ARVC-5 model was based on the ectopic expression of either the wild type (Tmem43^wt^) or the clinically relevant mutant (Tmem43^p.S333L^) (Klinke et al., 2022). In this study, we generated CRISPR-Cas9-mediated knock-in flies that hold the clinically relevant point mutation p.S333L in the *Tmem43* gene at its endogenous locus. Resulting animals are homozygous viable and, at least under laboratory conditions (i.e. without external stress), heart rate and heart rhythmicity were not significantly affected, compared to control animals. However, other characteristic Tmem43^pS333L^ overexpression phenotypes, e.g. reduced life span, reduced size and weight, or increased lipid droplet volume in fat cells (Klinke et al., 2022; Jürgens et al., 2025), were also observed in the knock-in model. Thus, the new Tmem43^p.S333L^ knock-in line replicates key phenotypes of the previous overexpression model while providing wild-type tissue and stage-specific expression of the mutant protein controlled by the endogenous promoter. Furthermore, the viability of hetero- and homozygous carrier flies allowed us to evaluate possible dosage effects of the mutation.

## RESULTS

### CRISPR/Cas9 mediated generation of Tmem43^p.S333L^ knock-in transgenic *Drosophila*

**Fig. 1.**
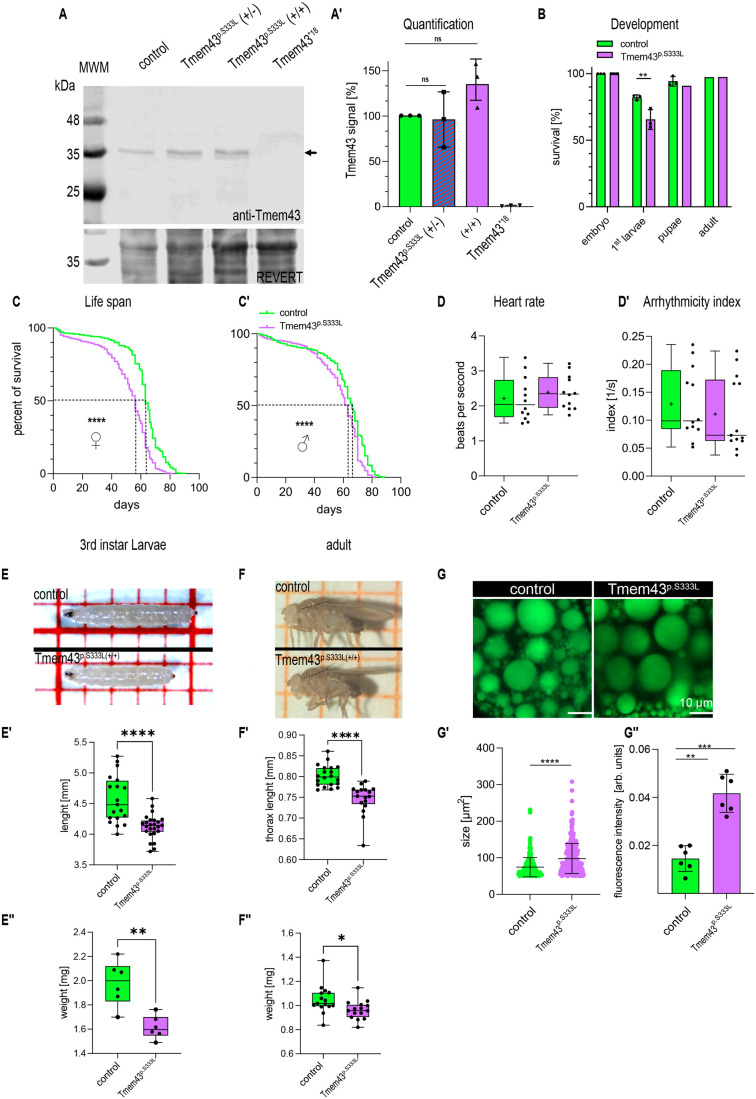
**Characterisation of a Tmem43^p.S333L^ knock-in line.** (A,A′) Western blot analyses detecting Tmem43 protein amounts under heterozygous p.S333L knock-in conditions, homozygous knock-in conditions, and wild-type conditions (control). Tmem43 amounts are comparable in all genetic backgrounds tested (arrow). A Tmem43 knockout mutant (Tmem43^*18^, Klinke et al., 2022) was co-analysed to confirm the specificity of the applied anti-Tmem43 antibody. REVERT-based total protein staining was used as a loading control. (B) During development, homozygous Tmem43^p.S333L^ 1st instar larvae (n=342) show a higher lethality compared to control animals (n=303). Other developmental stages are not affected by the mutation. (C,C′) The median and maximal life span of females (control, *n*=450; knock-in, *n*=605) and males (control, *n*=565; knock-in, *n*=549) expressing the mutant protein are reduced compared to the control. Median survival (f)=63 versus 56 days, maximum survival (f)=91 versus 82 days, median survival (m)=66 versus 63 days, maximum survival (m)=89 versus 82 days. (D,D′) Heart parameters, such as heart rate and arrhythmicity index, are not affected in 5-week-old males carrying the point mutation. (E) Homozygous Tmem43^p.S333L^ wandering 3rd instar larvae are significantly decreased in size (E′) and weight (E′′). (F) Homozygous Tmem43^p.S333L^ adult animals (males) are significantly decreased in size (F′) and weight (F′′). The dashed lines in F indicate the position of measurement. (G) Lipid droplet size is significantly increased in homozygous Tmem43^p.S333L^ wandering 3rd instar larvae. At least ten larvae per genotype were analysed. Quantification of BODIPY staining reveals that, in addition to an enlarged size (G′), corresponding droplets also exhibit an increased fluorescence intensity (G′′).

CRISPR/Cas9-mediated genome editing was applied to introduce the ARVC-5 relevant p.S333L missense mutation into the *Tmem43* gene locus. *Drosophila* lines carrying the *Tmem43* mutation were initially cultured as heterozygous balanced stocks. Presence of the missense mutation was verified by sequencing. We found that homozygous mutant animals are viable under laboratory breeding conditions. Therefore, we used hetero- and homozygous mutants for our experimental analyses (Fig. 1).

First, we analysed the expression level and stability of the mutant Tmem43^p.S333L^ protein in knock-in flies. Total protein extracts from complete specimens were probed with anti-Tmem43 antibodies. We found that the expression level was comparable to the expression of the wild-type protein in control animals (Fig. 1A,A′). This suggested that Tmem43^p.S333L^ is a stable protein that is not degraded prematurely by quality control mechanisms of the expressing cells.

Next, we analysed the Tmem43^p.S333L^ knock-in flies for developmental and physiological phenotypes (Fig. 1B,C). Survival rate and life span were likewise affected in Tmem43^p.S333L^ knock-in mutant females and males, similar to what we had observed in animals in which Tmem43^p.S333L^ was ubiquitously overexpressed (Klinke et al., 2022). Both the median and maximum life spans of females and males were reduced in animals expressing the mutant protein, compared to the control group (Fig. 1C). However, male flies overexpressing the mutant form of the protein, specifically in heart cells, exhibited arrhythmia (Klinke et al., 2022), whereas the knock-in mutants did not show any significant impact on heart performance under non-stress laboratory conditions (Fig. 1D). Since we did not observe any significant differences between male and female flies, all further experiments were conducted regardless of gender.

In line with the overexpression, knock-in larvae expressing the mutant Tmem43^p.S333L^ protein were significantly smaller than age-matched wild-type larvae (Fig. 1E,E′). The same phenotype was observed for the corresponding adult animals (Fig. 1F,F′). Consistent with the reduced size, both knock-in larvae (Fig. 1E′′) and adults (Fig. 1F′′) weighed significantly less than their respective wild-type controls. Moreover, Bodipy-stained lipid droplet size increased in the mutants compared to wild-type controls (Fig. 1G). Quantification of the BODIPY signals revealed that knock-in larvae store more lipids than the control group (Fig. 1G). These phenotypes are consistent with our observations in animals overexpressing the mutant Tmem43^p.S333L^ (Klinke et al., 2022).

### Tmem43^p.S333L^ causes accumulation of degenerated mitochondria in muscle tissue

**Fig. 2.**
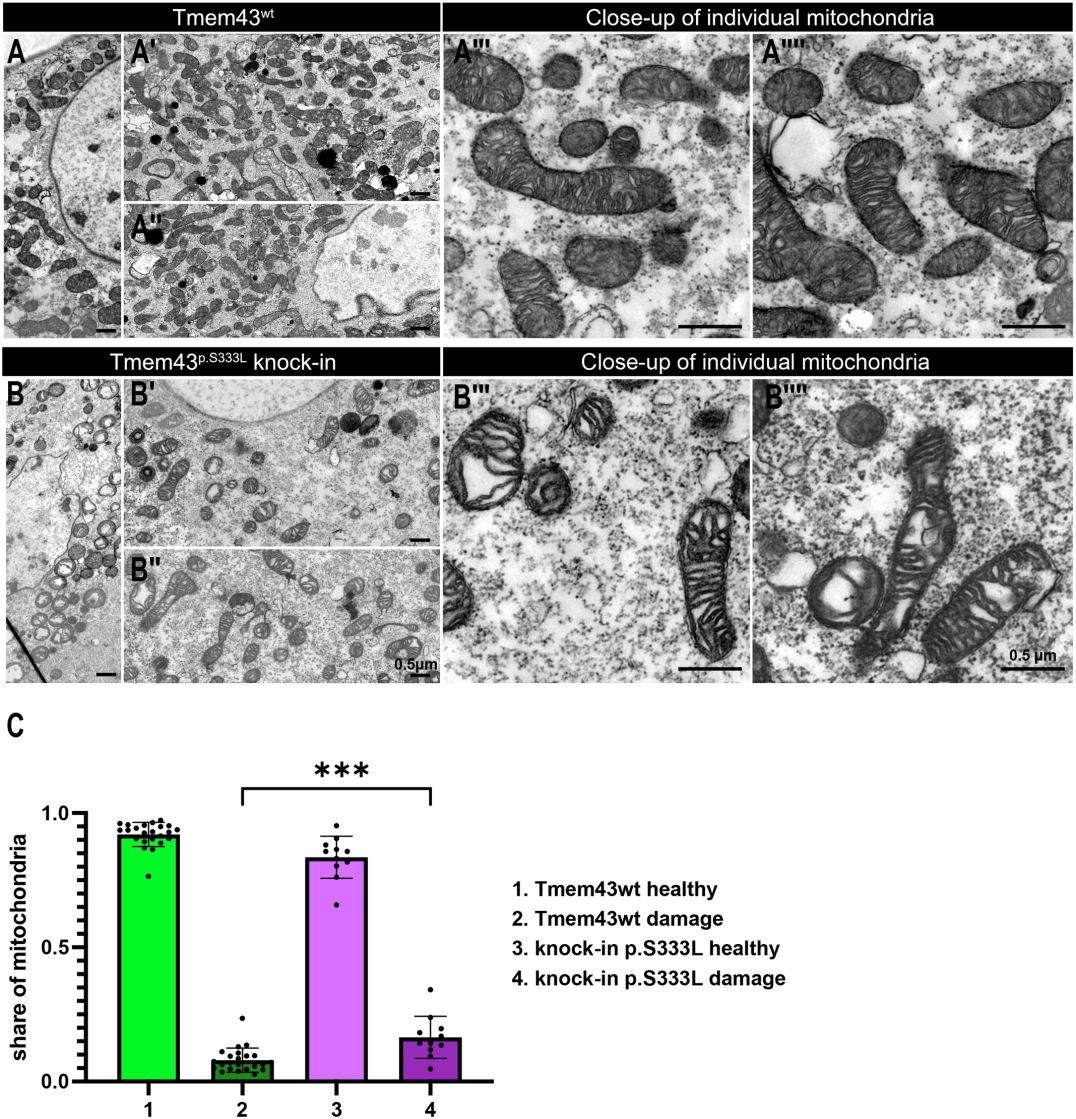
**The Tmem43^p.S333L^ knock-in line exhibits morphological defects in mitochondria.** (A) Representative TEM images of body wall musculature from control *Drosophila* samples (Tmem43wt). (A′–A″″) Different orientations and close-ups of individual mitochondria are shown (insets). (B) Representative TEM images of body wall musculature from transgenic Tmem43^p.S333L^ knock-in *Drosophila* samples. (B′–B″″) Different orientations and close-ups of individual mitochondria are shown (insets). (C) Quantification of damaged mitochondria for the different genotypes reveals significantly increased amounts of damaged mitochondria in mutant tissue (unpaired *t*-test, ***P<0.001). Under wild-type conditions, 92% healthy and 8% damaged mitochondria are present. For mutants, 84% of the mitochondria are classified as healthy, while the remaining 16% exhibit different manifestations of damage, including swelling, cristae degradation, and bursting. At least five animals per genotype were analysed.

We recently demonstrated that the expression of the mutant Tmem43^p.S333L^ protein dysregulates mitochondrial energy metabolism. This manifests as morphologically damaged mitochondria, as demonstrated in ultrastructural analyses of a patient's heart and in the *Drosophila* model (Jürgens et al., 2025). The mitochondria of muscle cells exhibit disturbed structure and impaired cristae formation, leading to swollen and ruptured mitochondria, as confirmed by transmission electron microscopy (TEM) (Jürgens et al., 2025). To evaluate these overexpression results under endogenous conditions in Tmem43 knock-in flies, we performed corresponding TEM ultrastructural analyses of wild-type and Tmem43^p.S333L^ knock-in body wall muscles (Fig. 2). When applying the same morphological criteria to evaluate mitochondrial fitness, we classify 8% of mitochondria in the wild type as damaged. This might reflect the continuous normal turnover of mitochondria. In Tmem43^p.S333L^ knock-in mutants, however, we find 16% damaged mitochondria. Thus, the knock-in mutants resembled the previously described overexpression phenotype of mitochondrial swelling, outer membrane rupture, intramitochondrial vacuolisation, and degradation of cristae structures, albeit to a milder extent (Jürgens et al., 2025).

### Comparative analysis of the proteome of heterozygous and homozygous knock-in and Tmem43^p.S333L^ overexpression animals

**Fig. 3.**
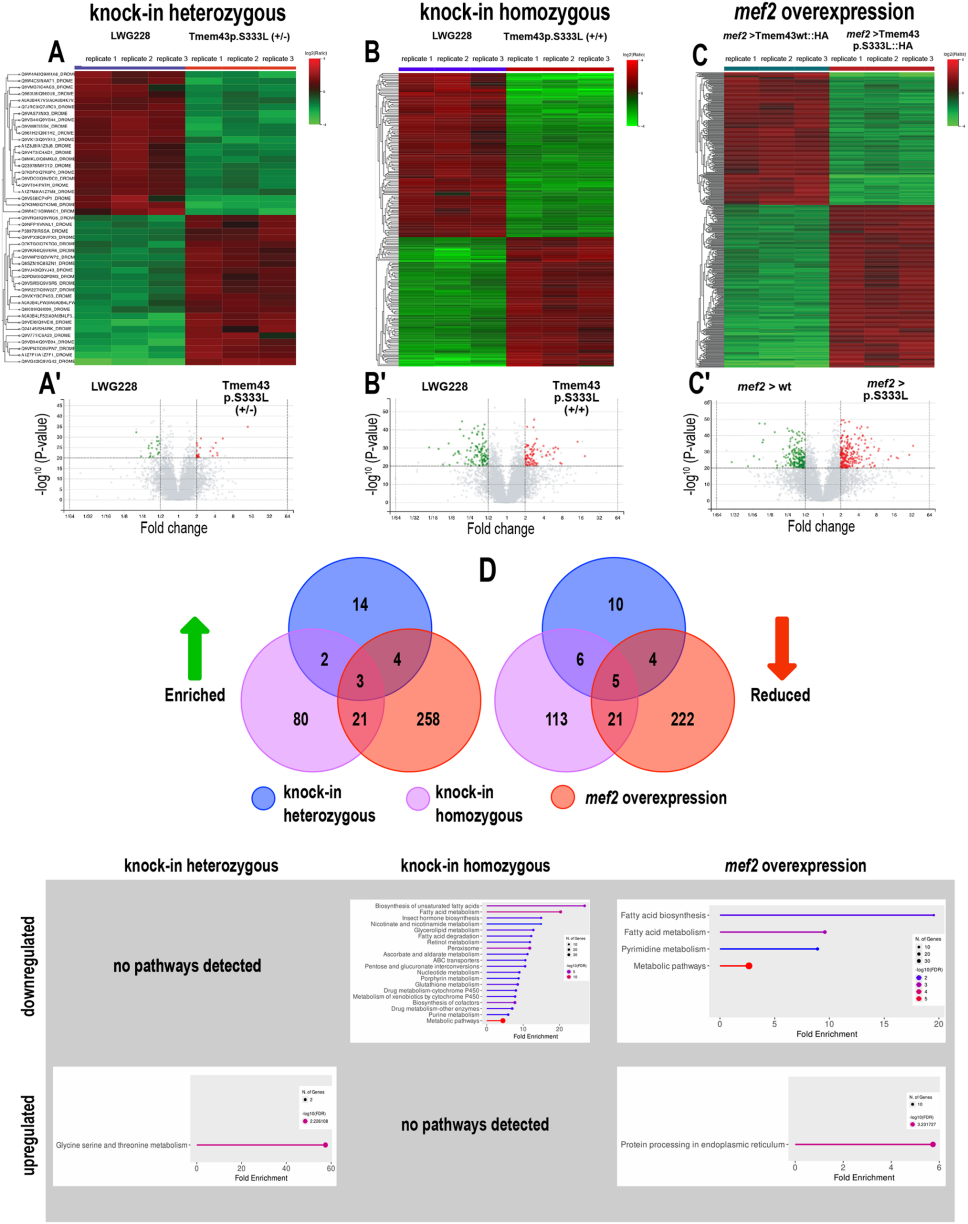
**Comparative proteomics reveal gene-dosage effects between the Tmem43^p.S333L^ knock-in lines and overexpression constructs.** (A–C) Heatmaps showing hierarchical clustering of proteins with significantly altered amounts in 3rd instar larvae heterozygous for Tmem43^p.S333L^ (A), homozygous for Tmem43^p.S333L^ (B), or *mef2*-Gal4-driven overexpression of Tmem43^p.S333L^::HA (C), relative to the indicated controls. (A′–C′) Volcano plots of the heatmaps depicted in A–C. Altered proteins are indicated in green (increased in control) and red (increased in Tmem43^p.S333L^). The comparisons are shown based on fold change (x-axis) and adjusted *P*-value (y-axis). (D) Venn diagrams illustrating the overlap of significantly enriched (left) and reduced (right) proteins across all datasets. (E) KEGG pathways (proteome-based) enriched or reduced in the depicted genetic backgrounds, relative to wild-type controls.

To better understand the knock-in effects at the molecular level, we compared the proteome composition of heterozygous knock-in, homozygous knock-in, and overexpression animals (Fig. 3). A compilation of all significantly altered proteins is depicted in Table S1. We found that the mutation's impact was dose-dependent. While heterozygous knock-in animals showed a relatively mild phenotype, with only a few proteins exhibiting significantly increased or reduced levels, homozygous animals were characterised by considerably stronger effects (48 proteins were significantly altered in heterozygous animals compared to 251 in homozygous animals). This effect was even more pronounced in Tmem43^p.S333L^ overexpression animals, with 538 proteins significantly altered (Fig. 3D).

Among the 48 proteins significantly altered in heterozygous knock-in animals, we identified several cell–cell junction proteins as being reduced. One was the smooth septate junction protein Snakeskin (Ssk), which was reduced to 35%. The other was Innexin 3 (Inx3), a gap junction protein that was reduced to 33% (Fig. 3). Significantly altered protein levels were also evident for three Cytochrome P450 (CYP4) family members, which are involved in diverse metabolic processes, including the ω-oxidation of fatty acids (Zhou et al., 2023). The proteins Cyp4p1, Cyp4ac3, and Cyp4ad1 were reduced to 24%, 38% and 41%, respectively. At the same time, increased levels of Cyp6a23 (374%) and Cyp4s3 (214%) were observed (Fig. 3). Given that the proteome of heterozygous mutant animals exhibited the mildest changes compared to controls, with 25 reduced and 23 increased proteins, KEGG pathway analyses identified only one significantly affected pathway (Fig. 3): altered glycine, serine, and threonine metabolism. The basis for this was increased levels of the proteins Glycine N-methyltransferase (Gnmt, 1420%) and Aminomethyltransferase (CG6415, 207%, Table S1).

**Fig. 4.**
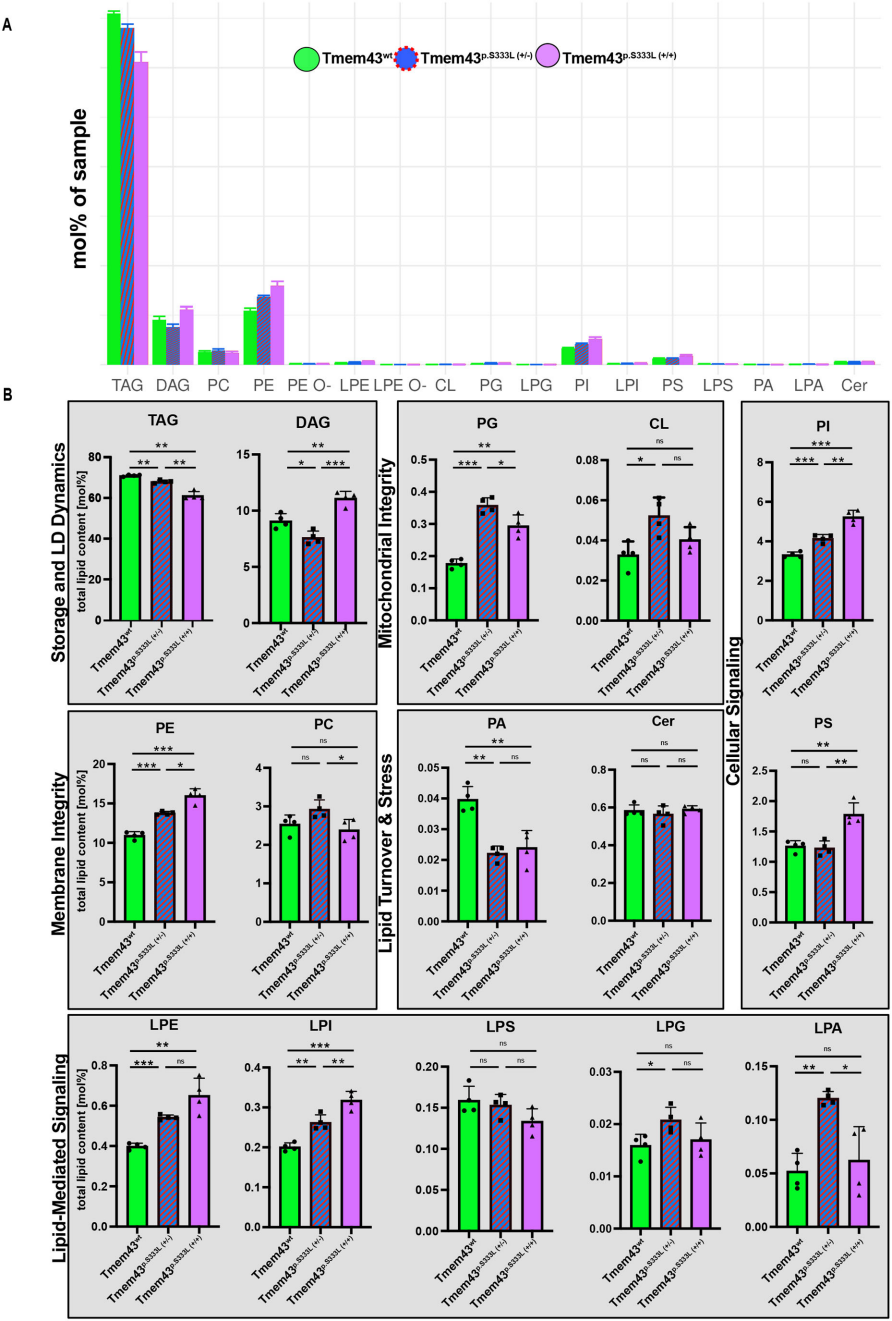
**Lipidomic analyses reveal alterations in lipid composition in Tmem43^p.S333L^ knock-in lines.** (A) Global lipid class distribution in 3rd instar larvae from wild-type (green), heterozygous Tmem43^p.S333L^ (blue), and homozygous Tmem43^p.S333L^ (purple) knock-in flies as determined by quantitative lipidomics. Data are expressed as mol% of total lipid content. Major lipid classes are grouped according to their functional roles: storage and LD dynamics (TAG, DAG), membrane integrity (PC, PE), mitochondrial membrane components (PG, CL), lipid turnover (PA, Cer), and signalling lipids (PI, PS, LPE, LPI, LPS, LPG, LPA). (B) Bar graphs showing quantifications for individual lipid classes categorised by biological function. Notably, TAG levels are significantly reduced in mutants, indicating impaired neutral lipid storage. PE and PG are significantly elevated in mutants, suggesting altered membrane composition and mitochondrial phospholipid remodelling. The accumulation of PI, PS, LPE, and LPI in homozygous animals reflects upregulation of signalling lipids. LPA and PA show dosage-dependent patterns, while CL, Cer, and LPS remain largely unchanged. Statistical significance was assessed using one-way ANOVA. For each genotype, four individual replicates, each consisting of ten 3rd instar larvae, were analysed. Data represent mean±s.e.m.; *P<0.05, **P<0.01, ***P<0.001, ****P<0.0001.

The proteome of homozygous mutant animals showed extensive changes compared to the control, with 145 proteins exhibiting reduced levels and 106 showing increased levels (Fig. 3D). Here, the KEGG pathway analysis identified multiple pathways as being affected (Fig. 3E). Several proteins essential for fatty acid (FA) metabolism were decreased, including those involved in FA synthesis, elongation, desaturation, activation, and ß-oxidation. The acetyl-CoA-carboxylase (ACC), which catalyses the ATP-dependent carboxylation of acetyl-CoA to malonyl-CoA, and thus the rate-limiting step of FA synthesis, was reduced to 35%. Other proteins with reduced amounts were Desaturase 1 (Desat1, reduced to 19%), which synthesises monounsaturated FAs, Baldspot (46%), which is the rate-limiting enzyme of the long-chain FA elongation cycle, and the FA activating enzymes Acsl (39%) and Bubblegum (Bgm, 33%). The levels of peroxisomal proteins were also decreased, including acyl-Coenzyme A oxidase 57D-d (Acox57D-d, reduced to 16%) and Peroxisomal multifunctional enzyme type 2 (Mfe2, 45%), which are involved in peroxisomal ß-oxidation. Additionally, peroxisomal membrane proteins and transporters, including Peroxisomal Membrane Protein 70 (Pmp70, 34%) and Pmp34 (29%), were reduced, which was also the case for peroxisomal biogenesis factor 16 (Pex16, 39%), involved in peroxisomal organisation, and Pex11 (42%), predicted to be involved in peroxisome fission. These peroxisomal alterations were observed only in the homozygous mutant, not in heterozygous or overexpression animals. Glycerolipid metabolism was also severely affected, with several key enzymes downregulated. These were Glycerol kinase 2 (Gk2, 33%), Glycerol-3-phosphate acyltransferase 4 (GPAT4, 42%), 1-Acylglycerol-3-phosphate O-acyltransferase 3 (AGPAT3, 50%), and AGPAT4 (34%). The decreased levels of these proteins, which are involved in TAG synthesis, are consistent with the overall reduced TAG levels we identified in the lipidomic analyses (Fig. 4). Another affected pathway was the glutathione (GSH) metabolism. Not only was Glutamate-cysteine ligase catalytic subunit (Gclc), which catalyses the rate-limiting step of GSH synthesis, reduced (30%), but also Glutathione synthetase 2 (Gss2, 27%), which catalyses the final synthesis step. Additionally, levels of Glutathione S transferase E10 (GstE10, 36%), involved in cellular stress responses, and a glutathione gamma-glutamate hydrolase (encoded by CG17636, 27%) were reduced. The previously mentioned proteins include mitochondrial envelope proteins, namely Baldspot and Gk2. Downregulation was also observed for proteins of the mitochondrial respirasome [ND-B14.7 (40%), ND-B17 (44%), and NP15.6 (44%)], the alpha-ketoglutarate dehydrogenase complex (Nc73EF), and the mitochondrial envelope [Mpcp2 (45%), ND-B14.7, Baldspot, ND-B17, Gk2, NP15.6, Tom70 (49%)]. Notably, the last group of affected proteins indicates impaired mitochondrial function and integrity, providing a molecular explanation for the observed mitochondrial degeneration in the homozygous Tmem43^p.S333L^ knock-in body wall muscles (Fig. 2).

The proteome of flies overexpressing Tmem43^p.S333L^ showed the most alterations, compared to the control, with 252 reduced and 286 increased proteins (Fig. 3D). Several KEGG pathways were affected (Fig. 3E). These included the nuclear envelope and structural proteins. The amount of Lamin β-receptor was about 2-fold lower in the mutant samples. The Lamin β-receptor is an integral constituent of the nuclear membrane that tethers heterochromatin to the periphery of the nucleus and contains a sterol reductase in its transmembrane domain. The loss of Lamin β-receptor in the mutant suggests altered nuclear-lamina-chromatin contacts and potentially altered cholesterol metabolism at the nuclear membrane. Consistent with this finding, the nuclear pore protein Nup210, which is essential for muscle cell integrity, was reduced to 36% in mutants, further highlighting nuclear envelope abnormalities. Interestingly, changes in structural muscle proteins were also evident. Flies overexpressing TMEM43^p.S333L^ showed enriched levels of several myofibrillar proteins. Mutants had a 2.6-fold increase in Troponin-C and a 2.1-fold increase in Tropomyosin 2. Troponin-C is the calcium-binding subunit of the troponin complex that regulates actin-myosin interaction during contraction (Ohtsuki and Morimoto, 2008). Interestingly, we also identified several ERMCS-associated proteins as misregulated in the overexpression mutants (Fig. 3, Table S1). Mitofusin, an MFN2 ortholog called Marf in *Drosophila*, was drastically reduced in the corresponding flies. Mutant protein levels were almost 70% reduced compared to wild-type samples. Marf is a GTPase associated with the outer mitochondrial membrane (OMM) that mediates mitochondrial fusion and maintains intact mitochondrial morphology. Consistent with this, we found that the MICOS subunit QIL1 was reduced by 41% in the mutant. QIL1 is a critical component of the ERMCS, establishing a complex on the mitochondrial side, and is involved in cristae formation (Guarani et al., 2015). In addition to this, PGAM5, a mitochondrial phosphatase that dephosphorylates MFN2, was to 44% reduced in mutant flies. These findings strongly suggest a profound defect in the regulatory network of mitochondrial fusion–fission and quality control in mutant cells. A potential role of Tmem43^p.S333L^ in ER-mitochondrial tethering and lipid transfer is further supported by our finding that wild-type samples had almost double the amount of Vps13. This large intermembrane lipid transfer protein can connect organelles and ensures lipid transport at contact sites (Kumar et al., 2018). Similarly, oxysterol-binding protein was also significantly reduced in mutants. Regarding the metabolic protein profile of the samples, again, considerable differences were observed. Pyruvate dehydrogenase kinase (Pdk) was found to be reduced by almost 55% in mutant samples. Pdk inhibits the pyruvate dehydrogenase (PDH) complex via phosphorylation, which results in reduced conversion of pyruvate to acetyl-CoA, favouring glycolysis over glucose oxidation. This means that the mutant may not be able to inhibit PDH efficiently, leading to increased acetyl-CoA levels that can be shuttled into the mitochondrial TCA cycle, thus promoting oxidative phosphorylation. The increased conversion of pyruvate to acetyl-CoA should reduce cellular pyruvate levels, which may explain why lactate dehydrogenase (LDH), which converts pyruvate to lactate under anaerobic conditions, was also significantly reduced in the mutant samples (Table S1). These results further indicate that compromised mitochondrial energy metabolism is a major impairment in Tmem43^p.S333L^ mutants.

### Comparative analysis of the lipidome of heterozygous and homozygous Tmem43^p.S333L^ knock-in animals

Tissues with high energy consumption, such as muscles including cardiomyocytes, can break down triglycerides (TAGs, lipids) into fatty acids through lipolysis and further utilise them for energy production via mitochondrial ß-oxidation. Our previous data using the fly model (Klinke et al., 2022; Jürgens et al., 2025), hiPSC-derived cardiomyocytes, and cardiac tissue from ARVC-5 patients (Ratnavadivel et al., 2026) revealed an altered energy metabolism in the respective Tmem43/TMEM43 mutants. Consequently, we analysed whether such effects are also present in the new *Drosophila* Tmem43 S333L knock-in mutants. Again, we compared heterozygous and homozygous mutants to control animals.

**Fig. 5.**
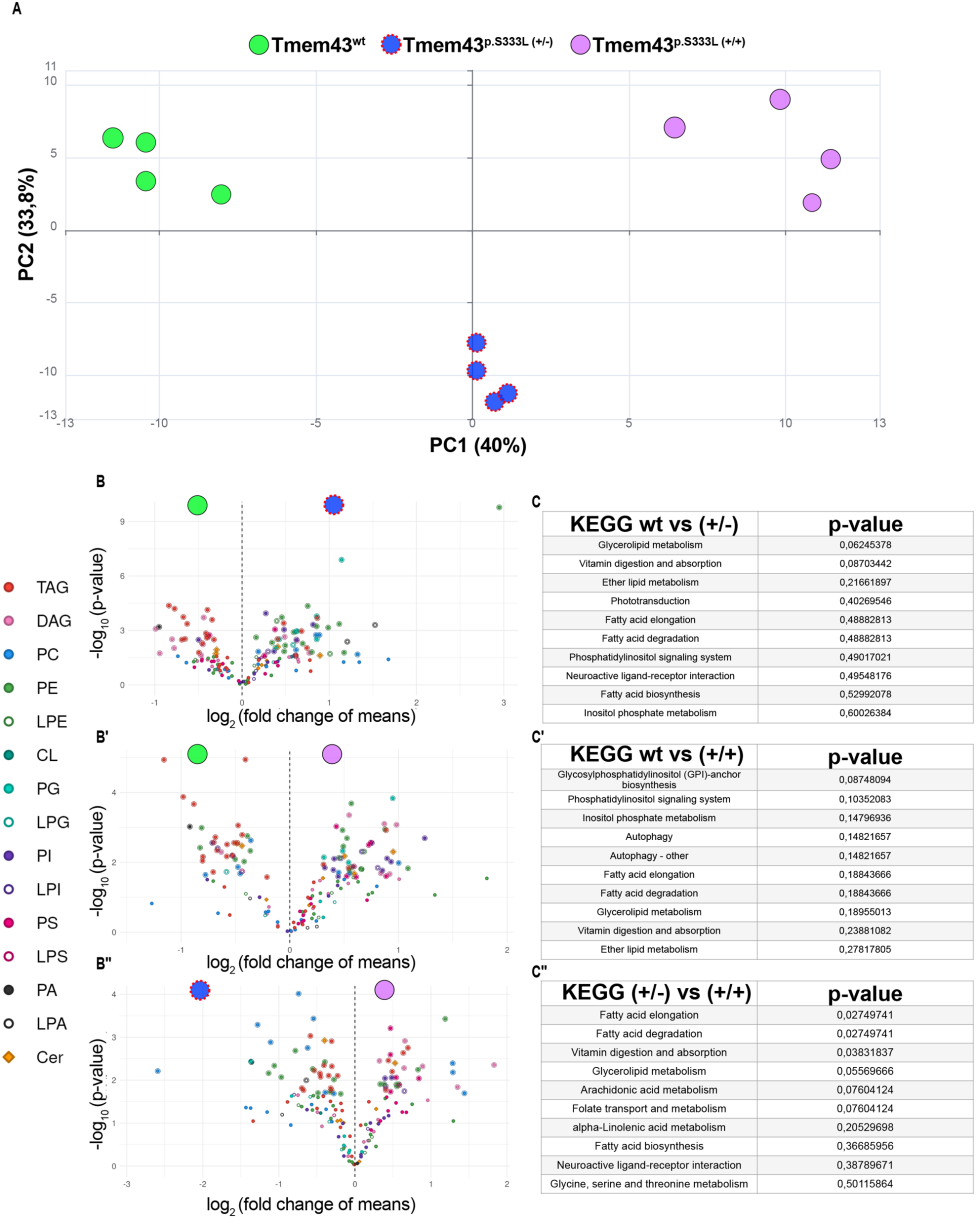
**Lipidomic analyses identify misregulated cellular processes in Tmem43^p.S333L^ knock-in lines.** (A) PCA of global lipidomic profiles from wild-type (green), heterozygous Tmem43^p.S333L^ (blue), and homozygous Tmem43^p.S333L^ (purple) 3rd instar larvae. A clear genotype-specific separation is evident. (B–B″) Volcano plots visualising pairwise comparisons of lipid species between genotypes. Data are represented as log2 fold change (x-axis) and -log10 *P*-value (y-axis). (B) Wild type versus heterozygous; (B′) wild type versus homozygous; (B″) heterozygous versus homozygous. Individual lipid species are colour-coded by class, highlighting consistent increases in phospholipids and reductions in neutral lipids as Tmem43^p.S333L^ dosage increases. (C–C″) KEGG pathway enrichment analyses based on significantly altered lipids from the comparisons depicted in B–B″. Functional clustering reveals pathways related to glycerolipid metabolism, fatty acid turnover, and phosphoinositide signalling.

In contrast to hiPSCs, we here analysed the consequences of the relevant Tmem43 mutation in a tissue and whole animal context. We performed comparative lipidomic profiling of wild-type and Tmem43^p.S333L^ flies, including both hetero- and homozygous conditions, to identify differences in the relative abundance of major lipid classes (Figs 4,5; Fig. S1). We found that the knock-in mutants exhibited a distinct shift in lipid composition, compared to the wild-type animals. In particular, changes in neutral lipid levels were observed alongside disturbances in certain phospholipid classes, which are crucial for membrane structure and organelle function.

TAG levels were reduced by 3 mol% in heterozygotes and 10 mol% in homozygous Tmem43^p.S333L^ mutant flies, relative to controls (Figs 4,5). TAG species accounted for about 80% of the total lipid content in the samples. In contrast, diacylglyceride (DAG) levels increased significantly in the homozygous samples (3 mol%), whereas the heterozygous flies showed a slight drop in DAG levels (1.5 mol%), compared to the control. Thus, loss of one Tmem43 allele causes a slight reduction in DAG, whereas loss of both wild-type alleles causes an overshoot in DAG species. Despite DAG accumulation in homozygotes, TAG levels were significantly reduced, suggesting a misregulation of neutral lipid storage or utilisation.

Furthermore, several membrane phospholipids were enriched in Tmem43^p.S333L^ knock-in flies. Among these, phosphatidylethanolamine (PE) and phosphatidylinositol (PI) levels exhibited a clear gene-dosage effect. PE levels were increased by 3 mol% in heterozygous mutants and by 5 mol% in homozygous mutants, while PI levels were increased by 1 mol% in heterozygous and by 2 mol% in homozygous samples. Phosphatidylglycerol (PG), a phospholipid associated with mitochondria, was also significantly altered in both mutant genotypes (control: 0.2 mol%; heterozygous: 0.4 mol%; homozygous: 0.3 mol%). Interestingly, cardiolipin (CL) levels, which are an indicator of mitochondrial integrity, remained unchanged. There was a difference in heterozygous animals, yet this effect was absent in the homozygous mutants. In line with the largely unchanged CL levels, phosphatidylcholine (PC), the most abundant phospholipid in cellular membranes, also did not show any significant differences (Fig. 4).

Phosphatidic acid (PA), a critical lipid intermediate in different lipid biosynthesis processes, was drastically reduced in both mutant genotypes (control: 0.04 mol%; heterozygous: 0.02 mol%; homozygous: 0.02 mol%). Phosphatidylserine (PS) levels were significantly elevated only in homozygous samples, whereas the wild-type and heterozygous flies exhibited comparable PS levels (control: 1.3 mol%; heterozygous: 1.2 mol%; homozygous: 1.8 mol%). This specific accumulation of PS in homozygous flies is notable because PS is usually synthesised in the ER and then transported to mitochondria for conversion into PE.

Regarding lysophospholipid levels, Tmem43^p.S333L^ mutants displayed considerable changes. Lysophosphatidylethanolamine (LPE) and lysophosphatidylinositol (LPI) were significantly increased in both hetero- and homozygous samples, again exhibiting a dose-dependent pattern (LPE control: 0.4 mol%; heterozygous: 0.5 mol%; homozygous: 0.7 mol%) (LPI control: 0.2 mol%; heterozygous: 0.3 mol%; homozygous: 0.3 mol%). These changes in LPE and LPI levels suggest enhanced phospholipid hydrolysis or remodelling in mutant flies.

In contrast, lysophosphatidylserine (LPS) showed a downward trend across all samples, but was not significantly altered. Interestingly, lysophosphatidic acid (LPA), a bioactive lipid intermediate, was selectively elevated in heterozygous flies only. Heterozygous LPA levels were two times higher than wild-type controls and homozygous samples (control: 0.05 mol%; heterozygous: 0.1 mol%; homozygous: 0.06 mol%). Ceramides (Cer), a group of sphingolipids involved in stress signalling and apoptosis, were not significantly altered.

KEGG-pathway analyses identified numerous processes that were similarly affected under both heterozygous and homozygous conditions relative to wild type. Among these were glycerolipid metabolism, vitamin digestion and absorption, ether lipid metabolism, FA elongation, FA degradation, phosphatidylinositol signalling, and inositol phosphate metabolism (Fig. 5C,C′), indicating a broad dysregulation of the FA metabolism as a main effect of the p.S333L mutation. Of note, among the similarly affected pathways, FA elongation, FA degradation, vitamin digestion and absorption, and glycerolipid metabolism exhibited a significant dose-specific response, with stronger effects occurring in homozygous knock-in larvae, relative to the heterozygous animals (Fig. 5C′′).

Collectively, these results demonstrate that the Tmem43^p.S333L^ mutation caused substantial perturbations in the *Drosophila* lipid homeostasis. Both neutral lipid storage and membrane lipid components were affected, with several lipid classes showing significant changes relative to the wild-type control. Although this was not true for all analysed lipid types, the observation that many lipid concentrations were more severely affected under homozygous conditions than under heterozygous conditions (Fig. 4) suggests a dose-dependent effect of the Tmem43^p.S333L^ mutation.

## DISCUSSION

### Hetero- and homozygous Tmem43^p.S333L^ knock-in mutants exhibit dose-specific effects

Comparative proteomic and lipidomic analyses revealed clear effects of the Tmem43^p.S333L^ mutation on the composition of the *Drosophila* proteome and lipidome, with stronger effects observed in homozygous conditions than in heterozygous conditions (Figs 3–5). This suggests that higher mutant protein load, especially in muscles, as confirmed by overexpression of the Tmem43^p.S333L^ variant exclusively in this tissue (Fig. 3), correlates with more severe cellular dysfunction. In heterozygous knock-in mutants, the residual wild-type Tmem43 might partially compensate for the effects of the mutation. This possibility is supported by the result that overexpression of wild-type TMEM43 improves the pathological phenotype in a mouse model of ARVC-5 (Lalaguna et al., 2025). However, once the wild-type protein is absent or subdued by mutant overexpression, the molecular and cellular impact of the mutation is significantly increased. This effect was particularly pronounced in lipidome analyses, in which many lipid species showed a clear dose-dependent response (e.g. TAG, PI, PE, LPE, LPI, Fig. 4). Of note, despite differences in the extent of alteration, KEGG-pathway analyses identified several processes that were similarly affected under both heterozygous and homozygous conditions. These pathways included glycerolipid metabolism, ether lipid metabolism, FA elongation, FA degradation, phosphatidylinositol signalling, and inositol phosphate metabolism (Fig. 5), indicating a broad dysregulation of the FA metabolism as a main effect of the Tmem43^p.S333L^ mutation. This dysregulation is likely reflected, amongst others, in the significant reduction in TAG levels that we observed, particularly in homozygous mutants (Fig. 4).

At the protein level, a similar relation to mutant copy number was observed. While under heterozygous conditions, 23 proteins were enriched and 25 reduced relative to the wild-type control; under homozygous conditions, the respective numbers were 106 and 145. Muscle-specific overexpression of Tmem43^p.S333L^ increased the numbers even more to 286 and 252, respectively. Regarding the latter approach, the restriction to muscle tissue may bias a comparison with results from heterozygous or homozygous knock-in animals. However, because ubiquitous overexpression of Tmem43^p.S333L^ caused larval/pupal lethality (Klinke et al., 2022), a more appropriate comparison was not possible at this stage. In addition, the global proteome analyses performed in this study serve as a starting point for further in-depth experimental work on the identified factors. Nevertheless, given the high reproducibility and low variance across individual biological replicates (Fig. 3A–C), the present dataset provides a valid basis for drawing initial conclusions about the physiological implications. Regarding affected pathways, again, processes involved in fatty acid metabolism were prominently represented in both homozygous knock-in and overexpression animals (Fig. 3), thus confirming the corresponding results from the lipidomic results (Fig. 5). Of note, failing hearts tend to downregulate fatty acid oxidation enzymes (Lionetti et al., 2011). In addition to fatty acids, nucleotide metabolism was significantly affected under both genetic conditions, further suggesting that disruptions in the FA-derived energy (ATP) supply are a main reason for the observed phenotypes in Tmem43^p.S333L^ animals. Of note, the alterations under heterozygous conditions did not reach a significance level that allowed identification of similar processes. This further supports dose-dependent effects. Moreover, the fact that the only pathway identified under heterozygous conditions – glycine, serine and threonine metabolism – was based on increased amounts of only two proteins (Gnmt, a glycine N-methyltransferase and CG6415, an aminomethyltransferase; Fig. S1) indicates rather limited effects in heterozygous animals. However, given the significant impact on lipid composition (Fig. 5C), the changes at the protein level under heterozygous conditions are still severe enough to cause similar physiological impairments as observed in homozygous animals (i.e. dysregulated FA metabolism, Fig. 5C,C′). This result is significant because the heterozygous state reflects the genetic condition in TMEM43 mutation carriers with the p. S358L mutation. Thus, corresponding flies could serve as a valuable model for further investigations into the molecular basis of human disease phenotypes.

### Increased Tmem43^p.S333L^ levels affect ERMCS composition

Another characteristic group of proteins that was altered in Tmem43^p.S333L^ overexpression animals is involved in endoplasmic reticulum-mitochondria contact site (ERMCS) formation and function. Key factors, including Marf, QIL1, PGAM5, Vps13, and OSBP, were significantly reduced in corresponding animals (Table S1). Consistent with the reduced amounts of these factors, we observed mitochondrial degeneration in both Tmem43^p.S333L^ overexpression animals (Jürgens et al., 2025) and homozygous p.S333L knock-in animals (Fig. 2). The uniformity of these results suggests that destabilisation or disruption of ERMCS formation or maintenance is a major cause of the p.S333L-induced phenotypes. In this regard, reduced concentrations of critical ERMCS components likely impair contact site stability and communication between the organelles. This directly affects lipid and ion exchange, ultimately compromising mitochondrial dynamics and energy production. An abnormal formation of ERMCSs in Tmem43^p.S333L^-overexpression animals has been shown previously (Jürgens et al., 2025). Moreover, the impaired ERMCS network in Tmem43^p.S333L^ may not only be responsible for the observed mitochondrial phenotypes but may also account for the downstream effects observed in proteomic and lipidomic profiling, as discussed above, and eventually for a shift in muscular energy metabolism. The downregulation of FA processing enzymes, such as Desat1, Acsl, and Bubblegum (Table S1), likely impairs ß-oxidation, thus inducing profound metabolic and energetic changes. This is consistent with our lipidomic data, which show a shift in lipid storage or utilisation (Fig. 4), somewhat reminiscent of the adipogenic remodelling reported for ARVC-5 (Lionetti et al., 2011).

### Of flies and humans

In our previous work, we utilised hiPSC-derived cardiomyocytes and human myocardial tissue from a TMEM43 wild-type and a p.S358L mutant transplant heart to demonstrate abnormal cardiac energy metabolism and impaired mitochondrial function and stability in human cells and tissues as a result of the mutation (Ratnavadivel et al., 2026). Similar effects were observed in a *Drosophila* model that overexpresses the malignant fly homologue Tmem43^p.S333L^ (Klinke et al., 2022; Jürgens et al., 2025).

In contrast to this previous model, the new knock-in line presented herein expresses the mutant construct under its native promoter, thereby maintaining physiological expression levels and avoiding overexpression artefacts. In the heterozygous state, the introduced point mutation also reflects the genetic state of human ARVC-5 patients carrying the heterozygous TMEM43^p.S358L^ mutation. Consistent with the overexpression model, the Tmem43^p.S333L^ knock-in induces mitochondrial degradation. Ultrastructural analyses reveal swollen, ruptured, and degraded organelles in *Drosophila* Tmem43^p.S333L^ knock-in (Fig. 2) and human TMEM43^p.S358L^ tissue (Jürgens et al., 2025). These shared defects are accompanied by perturbations in lipid homeostasis and utilisation.

We conclude that in both humans and the *Drosophila* model, TMEM43^p.S358L^ and Tmem43^p.S333L^ induce comparable cellular malfunctions. ER-mitochondrial contact site formation is affected by the respective mutations, leading to disrupted organelle communication. This results in abnormal lipid and energy metabolism, functionally defective mitochondria, and ultimately cardiomyocytes that lack the energy to maintain proper cell function. Based on these physiological and mechanistic similarities, we consider *Drosophila* a suitable and complementary model organism for ARVC-5 research. Despite the phylogenetic distance between flies and humans, we still observe conserved phenotypes that enable the investigation of the molecular effects of TMEM43^p.S358L^ in this model system, thereby leading to a more comprehensive understanding of the mechanisms underlying the aetiology of ARVC-5.

## MATERIALS AND METHODS

The *Drosophila* orthologue of the human Tmem43 gene was initially annotated as CG8111. However, we named the gene throughout the manuscript Tmem43 for easy reading.

### Fly stocks

The Tmem43^p.S333L^ knock-in line was generated using commercial services (see below). As control lines, LWG228 or *w*^1118^ were used. Overexpression was done using a UAS-Tmem43 construct described previously (Klinke et al., 2022).

### Generation of Tmem43^p.S333L^ knock-in flies

CRISPR-mediated mutagenesis was performed by WellGenetics Inc. (New Taipei City, Taiwan) according to (Kondo and Ueda, 2013). In brief, gRNA sequence GGCACCCGATCCTCAGTTCC[CGG] was cloned into U6 promoter plasmid(s). Cassette PBacDsRed, which contains 3xP3-DsRed flanked by PiggyBac (PBac) terminal repeats, and two homology arms with point mutation, was cloned into pUC57-Kan as a donor template for repair. Tmem43 targeting gRNAs and hs-Cas9 were supplied on plasmids, together with a donor plasmid for microinjection into embryos of the control strain LWG228. F1 flies carrying the selection marker (3xP3-DsRed) were further validated by genomic PCR and sequencing. Finally, the selection marker was excised by transposition of PBac. The injection strain was used as a control in subsequent experiments.

### Lifespan assay

The flies were collected 1–3 days after hatching, and males and females were separated. During the experiment, the flies were kept in cages in a climate chamber at 25°C and 65% humidity, on a 12-h dark/12-h light cycle. Animals received fresh food every 2–3 days. Dead flies were counted each time fresh food was offered. Flies sticking to the food were excluded from the experiment.

### Measuring the length and weight of the 3rd instar larvae

To immobilise and stretch the larvae, the animals were incubated in 60°C hot water for 2 s. Afterwards, animals were placed on a microscope slide, and pictures were taken. Subsequently, their length was calculated using Fiji (Schindelin et al., 2012). For adults, the thorax length was quantified in at least 15 3–5-day-old males as previously described (Lack et al., 2016). The thorax was measured from the base of the anterior humeral bristle to the posterior tip of the scutellum. For weight measurements, larvae or adults were grouped in cohorts of five and weighed. At least five cohorts per genotype were analysed.

### Lipid droplet analysis

3rd instar larvae were prepared to access the fat body. Tissue was fixed for 30 min in 4% formaldehyde with agitation, then washed several times with 1x PBS. Lipid droplets (LDs) were stained with BODIPY 493/503 (Invitrogen) for 30 min, with slight agitation and in darkness. After washing for 10 min, the tissue was embedded in Fluoromount/DAPI and analysed under a Zeiss LSM800. Images were captured with a Zeiss Apo 40x lens and analysed for LD number and size using Fiji image analysis software (Schindelin et al., 2012; Rueden et al., 2017). For statistical analyses, ten animals per genotype were stained and analysed.

To quantify the BODIPY signal, cohorts of ten larvae were collected and weighed. Afterwards, animals were cut open and stained with BODIPY 493/503 for 30 min. After washing with 1× PBS, samples were homogenised in 1% Triton X-100 in 1× PBS. Then, the samples were centrifuged for 1 min at 13,000 ***g*** to remove insoluble parts from the solution. Finally, absorption at 493 nm was measured with a NanoDrop spectrophotometer.

### Western blot

SDS-PAGE and western blotting were performed using standard protocols. The primary antibody used was rabbit anti-Tmem43 (1:100, Paululat Lab). The secondary antibody was IRDye^®^ 680 RD goat anti-rabbit (1:5000, LiCor, Lincoln, NE, USA). The membranes were imaged with an Odyssey Clx Imager (LiCor), and quantification was performed using Empiria Software (LiCor).

### Semi-automated optical heartbeat analysis

Characteristic heart parameters, including heart rate, heart period, systolic and diastolic intervals, fractional shortening, and rhythmicity, were measured using semi-automatic optical heartbeat analysis, SOHA (Ocorr et al., 2009). For all experiments, 5-week-old male flies were used.

### TEM

TEM imaging and analysis of mitochondria were performed as previously described (Klinke et al., 2022). In short, *Drosophila* 3rd instar larvae were cut open and prepared to reveal the body wall musculature, then fixed and embedded in Epon blocks. Individual slices around the nuclei were obtained by microtome sectioning. After further preparation for imaging, TEM panoramas of the relevant perinuclear regions were acquired. Images were analysed, and mitochondria were visually separated into healthy and damaged. Percentages for these categories were calculated for each panorama and then plotted using Prism 10 (GraphPad^®^).

### Lipidomics

Animals were prepared according to the sample preparation guidelines for Lipotype GmbH commercial services. For each genotype, four individual replicates were collected. The samples were sent on dry ice to Lipotype (Dresden, Germany). The Lipotype Zoom Pro app was used to filter the analysed data and evaluate the filtered datasets statistically. Data were filtered using an initial *P*-value cutoff of 0.05, a minimal fold change of 1.3x and the proportion of missing values allowed was set to 0.3 across individual cohorts (Fig. S1). This filtering reduced the initial amount of 685 values down to 183 remaining values that are part of the statistical analysis.

### Lipid extraction for mass spectrometry

Mass spectrometry-based lipid analysis was performed by Lipotype GmbH (Dresden, Germany) as previously described (Surma et al., 2015). Lipids were extracted using a chloroform/methanol procedure (Ejsing et al., 2009). Samples were spiked with internal lipid standard mixture containing: CL 14:0/14:0/14:0/14:0, Cer 18:1;2/17:0, DAG 17:0/17:0, hexosylceramide (HexCer) 18:1;2/12:0, LPA 17:0, lyso-phosphatidylcholine (LPC) 12:0, LPE 17:1, lyso-phosphatidylglycerol (LPG) 17:1, LPI 17:1, LPS 17:1, PA 17:0/17:0, PC 15:0/18:1 D7, PE 17:0/17:0, PG 17:0/17:0, PI 16:0/16:0, PS 17:0/17:0, cholesterol ester (CE) 16:0 D7, sphingomyelin (SM) 18:1;2/12:0;0, TAG 17:0/17:0/17:0. After extraction, the organic phase was transferred to an infusion plate and dried in a speed vacuum concentrator. The dry extract was re-suspended in 7.5 mM ammonium formiate in chloroform/methanol/propanol (1:2:4; V:V:V). All liquid-handling steps were performed using the Hamilton Robotics STARlet robotic platform with the Anti-Droplet Control feature for organic-solvent pipetting.

### Mass spectrometry for lipidomics

Samples were analysed by direct infusion on a QExactive mass spectrometer (Thermo Fisher Scientific) equipped with a TriVersa NanoMate ion source (Advion Biosciences). Samples were analysed in both positive and negative ion modes with a resolution of R_m/z=200_=280,000 for MS and R_m/z=200_=17,500 for MSMS experiments, in a single acquisition. MSMS was triggered by an inclusion list encompassing corresponding MS mass ranges scanned in 1 Da increments (Surma et al., 2015). Both MS and MSMS data were combined to monitor CE, DAG, TAG, and TAG O- ions as ammonium adducts; LPC, LPC O-, PC, and PC O- as formiate adducts; and CL, LPS, PA, PE, PE O-, PG, PI, and PS as deprotonated anions. MS only was used to monitor LPA, LPE, LPE O-, LPG, and LPI as deprotonated anions, and Cer, HexCer, and SM as formiate adducts.

### Data analysis and post-processing

Data were analysed using LipotypeXplorer, a proprietary software developed by Lipotype GmbH, based on LipidXplorer (Herzog et al., 2012, 2013). Data post-processing and normalisation were performed using an in-house developed data management system. Only lipid identifications with a signal-to-noise ratio >5 and a signal intensity 5-fold higher than in corresponding blank samples were considered for further data analysis.

### Sample preparation for proteomic analyses

Samples were prepared as described previously (Dehnen et al., 2020; Meyer et al., 2024). In brief, 3rd instar larvae were batch-frozen in cohorts of five and three biological replicates per genotype were analysed. Animals were collected in lysis buffer (iST sample preparation kit, PreOmics, Martinsried, Germany) and further processed for mass spectrometry analyses using the ‘iST Kit’ (PreOmics) according to the manufacturer's instructions. Briefly, solubilisation, reduction, and alkylation were performed in sodium deoxycholate (SDC) buffer containing TCEP and 2-chloroacetamide. Proteins were enzymatically hydrolysed overnight at 37°C by adding LysC and trypsin. Peptides were desalted, dried by vacuum centrifugation and reconstituted in 20 μl 0.1% trifluoroacetic acid.

### Mass spectrometry for proteomics

Dried peptides were resuspended in 10 µl LC-Load, resulting in a protein concentration of 100 ng/μl. 1 μl was used to perform reversed-phase chromatography on a Thermo Ultimate 3000 RSLC nano system connected to a TimsTOF HT mass spectrometer (Bruker Corporation, Bremen) through a Captive Spray Ion source. Peptides were separated on an Aurora Gen3 C18 column (25 cm×75 μm×1.6 μm) with CSI emitter (Ionoptics, Australia) at temperature of 40°C and eluted from the column via a linear gradient of acetonitrile from 10–35% in 0.1% formic acid for 44 min at a constant flow rate of 300 nl/min following a 7 min increase to 50%, and finally, 4 min to reach 85% buffer B. Eluted peptides were directly electro-sprayed into the mass spectrometer at an electrospray voltage of 1.5 kV and 3 l/min Dry Gas. The mass spectrometer settings of the timsTOF were adjusted to positive Ion polarity with a mass spectrometer range from 100 to 1700 m/z. The scan mode was set to DIA. The ion mobility was ramped from 0.7 V/cm^2^ to 1.5 V/cm^2^ over 100 ms. The accumulation time was also set to 100 ms. 10 PASEF ramps per cycle resulted in a duty cycle time of 1.17 s. The target intensity was adjusted to 14,000, the intensity threshold to 1200. The dynamic exclusion time was set to 0.4 min to avoid repeated scanning of precursor ions; their charge states were limited to 0–5.

The data were loaded into PeaksOnline Version 1.9 and analysed using the PeaksOnline workflow (DeNovo and DB Search) with a Precursor mass error tolerance of 15 ppm, Fragment Mass error tolerance of 0.5 Da, CSS Error tolerance of 0.05, and a Missed Cleavage of 2. As a modification, Carbamidomethylation (C) (fix) and Oxidation (M) (variable) were chosen.

As a protein database, the *Drosophila-*specific database (UP000000803, www.uniprot.org/proteomes/UP000000803) was used. The results were filtered for peptides with an FDR of 1%, for proteins by the −10lgP of 20. Classification as a possible interaction partner required *P*<0.05, with quantification based on at least two individual protein-specific peptides.

### Statistical analysis

For statistical analyses, Prism 10 (GraphPad, Boston, MA, USA) was used. Datasets were analysed for normal distribution using the D'Agostino & Pearson test. If normally distributed, the one-way ANOVA test was used. Otherwise, the Kruskal–Wallis test was applied. If only two datasets were compared, an unpaired *t*-test was done. Life-span data were analysed using log-rank (Mantel–Cox) tests.

For all tests, a *P*-value <0.05 was considered significant (*P<0.05, **P<0.01, ***P<0.001, ****P<0.0001). For boxplots, the center line of a plot indicates the median; the upper and lower bounds indicate the 75th and 25th percentiles, respectively; and the whiskers indicate the minimum and maximum. The proteomics data were statistically analyzed and visualized with PEAKS Studio software (v12.5, Bioinformatics Solutions Inc., Waterloo, Ontario, Canada). All experiments were performed at least in triplicates.

## Supplementary Material

10.1242/biolopen.062326_sup1 Supplementary information

Table S1.

## References

[BIO062326C1] Bengtsson, L. and Otto, H. (2008). LUMA interacts with emerin and influences its distribution at the inner nuclear membrane. *J. Cell Sci.* 121, 536-548. 10.1242/jcs.019281 18230648

[BIO062326C2] Dehnen, L., Janz, M., Verma, J. K., Psathaki, O. E., Langemeyer, L., Fröhlich, F., Heinisch, J. J., Meyer, H., Ungermann, C. and Paululat, A. (2020). A trimeric metazoan Rab7 GEF complex is crucial for endocytosis and scavenger function. *J. Cell Sci.* 133, jcs247080. 10.1242/jcs.247080 32499409

[BIO062326C3] Dreger, M., Bengtsson, L., Schoneberg, T., Otto, H. and Hucho, F. (2001). Nuclear envelope proteomics: novel integral membrane proteins of the inner nuclear membrane. *Proc. Natl. Acad. Sci. USA* 98, 11943-11948. 10.1073/pnas.211201898 11593002 PMC59747

[BIO062326C4] Ejsing, C. S., Sampaio, J. L., Surendranath, V., Duchoslav, E., Ekroos, K., Klemm, R. W., Simons, K. and Shevchenko, A. (2009). Global analysis of the yeast lipidome by quantitative shotgun mass spectrometry. *Proc. Natl. Acad. Sci. USA* 106, 2136-2141. 10.1073/pnas.0811700106 19174513 PMC2650121

[BIO062326C5] Gu, Q., Xu, F., Orgil, B. O., Khuchua, Z., Munkhsaikhan, U., Johnson, J. N., Alberson, N. R., Pierre, J. F., Black, D. D., Dong, D. et al. (2022). Systems genetics analysis defines importance of TMEM43/LUMA for cardiac- and metabolic-related pathways. *Physiol. Genomics* 54, 22-35. 10.1152/physiolgenomics.00066.2021 34766515 PMC8721901

[BIO062326C6] Guarani, V., McNeill, E. M., Paulo, J. A., Huttlin, E. L., Frohlich, F., Gygi, S. P., Van Vactor, D. and Harper, J. W. (2015). QIL1 is a novel mitochondrial protein required for MICOS complex stability and cristae morphology. *eLife* 4, e06265. 10.7554/eLife.06265 25997101 PMC4439739

[BIO062326C7] Herzog, R., Schuhmann, K., Schwudke, D., Sampaio, J. L., Bornstein, S. R., Schroeder, M. and Shevchenko, A. (2012). LipidXplorer: a software for consensual cross-platform lipidomics. *PLoS ONE* 7, e29851. 10.1371/journal.pone.0029851 22272252 PMC3260173

[BIO062326C8] Herzog, R., Schwudke, D. and Shevchenko, A. (2013). LipidXplorer: software for quantitative shotgun lipidomics compatible with multiple mass spectrometry platforms. *Curr. Protoc. Bioinformatics* 43, 14 12 11-14 12 30. 10.1002/0471250953.bi1412s43 26270171

[BIO062326C9] Jürgens, K. J., Menzel, L., Klinke, N., Schäper, L., Breitsprecher, L., Holtmannspötter, M., Psathaki, O. E., Walter, S., Ratnavadivel, S., Malmendal, A. et al. (2025). The ARVC-5-associated protein TMEM43 controls mitochondrial energy metabolism by stabilising ER-mitochondrial contact sites. *Cell. Mol. Life Sci.* 82, 400. 10.1007/s00018-025-05942-z 41236655 PMC12618784

[BIO062326C10] Klinke, N., Meyer, H., Ratnavadivel, S., Reinhardt, M., Heinisch, J. J., Malmendal, A., Milting, H. and Paululat, A. (2022). A *Drosophila melanogaster* model for TMEM43-related arrhythmogenic right ventricular cardiomyopathy type 5. *Cell. Mol. Life Sci.* 79, 444. 10.1007/s00018-022-04458-0 35869176 PMC9307560

[BIO062326C11] Kondo, S. and Ueda, R. (2013). Highly improved gene targeting by germline-specific Cas9 expression in *Drosophila*. *Genetics* 195, 715-721. 10.1534/genetics.113.156737 24002648 PMC3813859

[BIO062326C12] Kumar, N., Leonzino, M., Hancock-Cerutti, W., Horenkamp, F. A., Li, P., Lees, J. A., Wheeler, H., Reinisch, K. M. and De Camilli, P. (2018). VPS13A and VPS13C are lipid transport proteins differentially localized at ER contact sites. *J. Cell Biol.* 217, 3625-3639. 10.1083/jcb.201807019 30093493 PMC6168267

[BIO062326C13] Lack, J. B., Monette, M. J., Johanning, E. J., Sprengelmeyer, Q. D. and Pool, J. E. (2016). Decanalization of wing development accompanied the evolution of large wings in high-altitude *Drosophila*. *Proc. Natl. Acad. Sci. USA* 113, 1014-1019. 10.1073/pnas.1515964113 26755605 PMC4743785

[BIO062326C14] Lalaguna, L., Arevalo-Nunez de Arenas, M., Lopez-Olaneta, M., Villalba-Orero, M., Jimenez-Rioboo, R. J., Gomez-Gaviro, M. V., Isern, J., Munoz-Canoves, P., Byrne, B. J., Ochoa, J. P. et al. (2025). Overexpression of Wild-Type TMEM43 Improves Cardiac Function in Arrhythmogenic Right Ventricular Cardiomyopathy Type 5. *Circ. Res.* 136, 830-844. 10.1161/CIRCRESAHA.124.325848 40091736 PMC11984551

[BIO062326C15] Lionetti, V., Stanley, W. C. and Recchia, F. A. (2011). Modulating fatty acid oxidation in heart failure. *Cardiovasc.Res.* 90, 202-209. 10.1093/cvr/cvr038 21289012 PMC3078800

[BIO062326C16] Merner, N. D., Hodgkinson, K. A., Haywood, A. F. M., Connors, S., French, V. M., Drenckhahn, J.-D., Kupprion, C., Ramadanova, K., Thierfelder, L., McKenna, W. et al. (2008). Arrhythmogenic right ventricular cardiomyopathy type 5 is a fully penetrant, lethal arrhythmic disorder caused by a missense mutation in the TMEM43 gene. *Am. J. Hum. Gen.* 82, 809-821. 10.1016/j.ajhg.2008.01.010 PMC2427209 18313022

[BIO062326C17] Meyer, H., Bossen, J., Janz, M., Müller, X., Kunzel, S., Roeder, T. and Paululat, A. (2024). Combined transcriptome and proteome profiling reveal cell-type-specific functions of Drosophila garland and pericardial nephrocytes. *Commun. Biol.* 7, 1424. 10.1038/s42003-024-07062-z 39487357 PMC11530456

[BIO062326C18] Ocorr, K., Fink, M., Cammarato, A., Bernstein, S. and Bodmer, R. (2009). Semi-automated optical heartbeat analysis of small hearts. *J. Vis. Exp.* 31, pii: 1435. 10.3791/1435 PMC3150057 19759521

[BIO062326C19] Ohtsuki, I. and Morimoto, S. (2008). Troponin: regulatory function and disorders. *Biochem. Biophys. Res. Commun.* 369, 62-73. 10.1016/j.bbrc.2007.11.187 18154728

[BIO062326C20] Ratnavadivel, S., Dammeier, J., Gaertner, A., de Toledo, M. A. S., Zenke, M., Gummert, J., Bloch Rasmussen, T., Klinke, N., Jurgens, K., Meyer, H. et al. (2024). Generation of a TMEM43 knockout human induced pluripotent stem cell line (HDZi003-A-1) using CRISPR/Cas9. *Stem Cell Res.* 76, 103354. 10.1016/j.scr.2024.103354 38430734

[BIO062326C21] Ratnavadivel, S., Jürgens, K. J., Klinke, N., Gärtner, A., Groß, J., Klingel, K., Malmendal, A., Walter, S., Boen, H., Van Craenenbroeck, E., Schramm, R., Kostareva, A., Gummert, J., Kassner, A., Meyer, H., Paululat A. and Milting, H. (2026). Newfoundland Mutation TMEM43-p.S358L Causes Impaired Cardiac Energy Metabolism and Mitochondrial Function Through Altered Protein Interaction. *Circ Genom Precis Med*. e005171. 10.1161/CIRCGEN.125.005171 41919408

[BIO062326C22] Rueden, C. T., Schindelin, J., Hiner, M. C., DeZonia, B. E., Walter, A. E., Arena, E. T. and Eliceiri, K. W. (2017). ImageJ2: ImageJ for the next generation of scientific image data. *BMC Bioinform.* 18, 529. 10.1186/s12859-017-1934-z PMC5708080 29187165

[BIO062326C23] Schindelin, J., Arganda-Carreras, I., Frise, E., Kaynig, V., Longair, M., Pietzsch, T., Preibisch, S., Rueden, C., Saalfeld, S., Schmid, B. et al. (2012). Fiji: an open-source platform for biological-image analysis. *Nat. Methods* 9, 676-682. 10.1038/nmeth.2019 22743772 PMC3855844

[BIO062326C24] Surma, M. A., Herzog, R., Vasilj, A., Klose, C., Christinat, N., Morin-Rivron, D., Simons, K., Masoodi, M. and Sampaio, J. L. (2015). An automated shotgun lipidomics platform for high throughput, comprehensive, and quantitative analysis of blood plasma intact lipids. *Eur. J. Lipid Sci. Technol.* 117, 1540-1549. 10.1002/ejlt.201500145 26494980 PMC4606567

[BIO062326C25] Zhou, M., Li, J., Xu, J., Zheng, L. and Xu, S. (2023). Exploring human CYP4 enzymes: Physiological roles, function in diseases and focus on inhibitors. *Drug. Discov. Today* 28, 103560. 10.1016/j.drudis.2023.103560 36958639

