# Supplementary material for: An ARVC-5 Drosophila knock-in model reveals new functions of Tmem43 in lipid homeostasis
Source: Biol Open. 2026 Apr 15;15(4):bio062326. doi: 10.1242/bio.062326 (PMC13133764; doi:10.1242/bio.062326)
Supplement: Supplementary information [file biolopen-15-062326-s1.pdf]

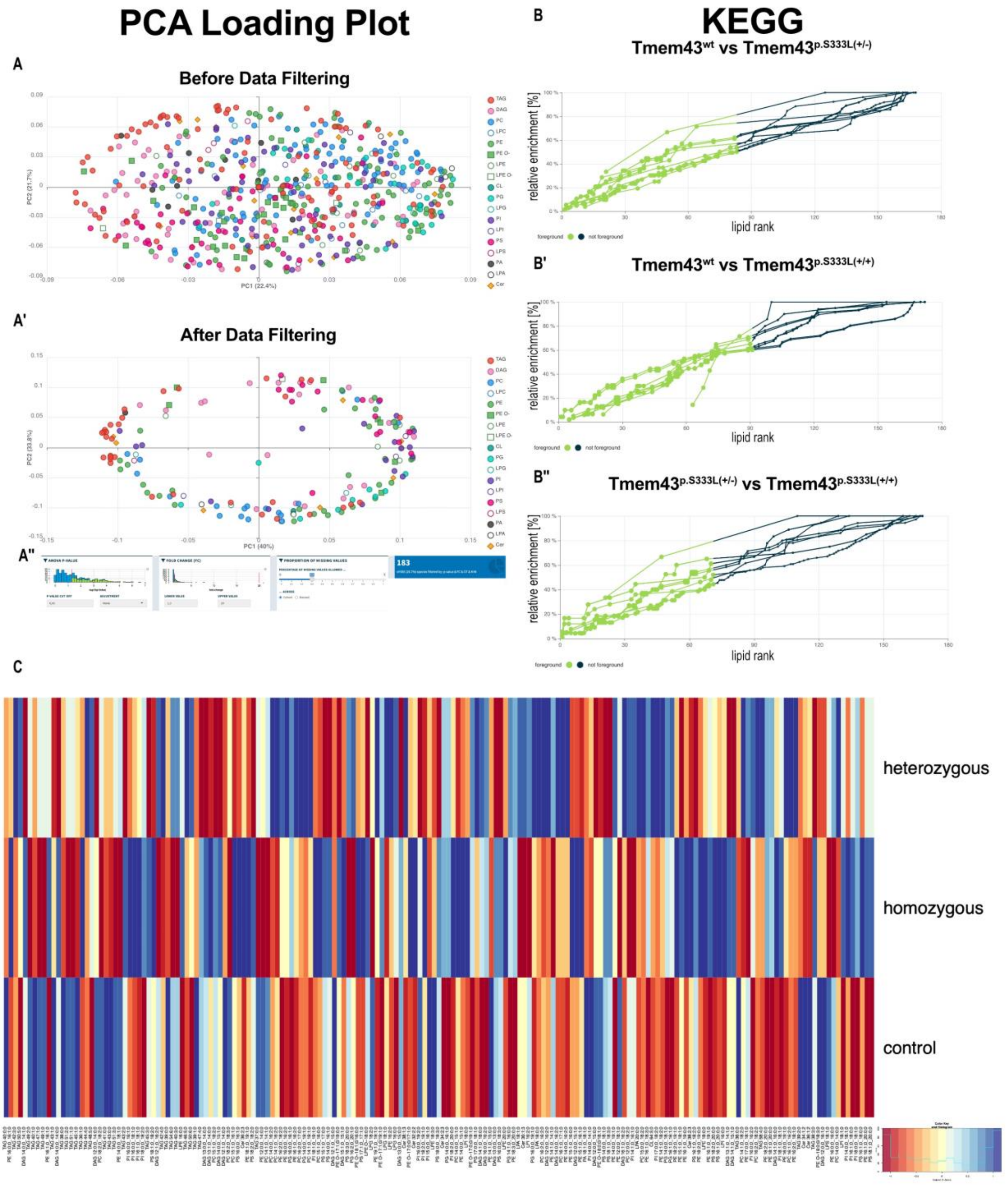

Fig. S1. Technical approach for Lipidomics data processing

Principal component analysis (PCA) before (A) and after (A') data filtering with LipotypeZoomPRO software (Lipotype, Dresden, Germany). The filtered datasets show a clearer separation of lipid species based on the parameters for data filtering (A''). Filtering conditions were a p-value threshold of p=0.05,

Biology Open • Supplementary information

fold change min=1.3 and max=20, and a maximum proportion of missing values across cohorts of 0.3. (B–B″) KEGG pathway enrichment analysis comparing Tmem43wt with heterozygous Tmem43$^{p.S333L}$ (B), Tmem43wt with homozygous Tmem43$^{p.S333L}$ (B‘), and heterozygous with homozygous mutants (B‘‘). Enrichment score plots indicate whether lipids belonging to a KEGG pathway, as indicated in Fig. 5, are significantly enriched at the top or bottom of the ranked list of differential lipids. Green dots represent pathway-associated lipids; black dots indicate the enrichment profile. (C) Heatmap representation of lipid species abundance across control, heterozygous, and homozygous Tmem43$^{p.S333L}$ knock-in samples. Rows correspond to lipid classes and individual species, colour-coded by relative abundance.

Table S1.

Available for download at
https://journals.biologists.com/bio/article-lookup/doi/10.1242/bio.062326#supplementary-data
